# Continuous Glucose Monitoring: A Possible Aid for Detecting Hypoglycemic Events during Insulin Tolerance Tests

**DOI:** 10.3390/s23156892

**Published:** 2023-08-03

**Authors:** Soo Yeun Sim, Moon Bae Ahn

**Affiliations:** Department of Pediatrics, College of Medicine, The Catholic University of Korea, Seoul 06591, Republic of Korea; sooyeunsim8@gmail.com

**Keywords:** hypopituitarism, glucose-monitoring system, hypoglycemia, growth hormone deficiency

## Abstract

The combined pituitary function test evaluates the anterior pituitary gland, while the insulin tolerance test evaluates growth hormone deficiencies. However, successful stimulation requires achieving an appropriate level of hypoglycemia. Close medical supervision for glucose monitoring is required during hypoglycemia induction and the test is often very tedious. In addition, a capillary blood sugar test (BST) and serum glucose levels may differ greatly. An alternative approach may be utilizing a continuous glucose-monitoring (CGM) system. We provide three cases in which CGM was successfully used alongside a standard BST and serum glucose levels during the combined pituitary function test to better detect and induce hypoglycemia. Three participants who were diagnosed with multiple pituitary hormone deficiencies during childhood were re-evaluated in adulthood; a Dexcom G6 CGM was used. The CGM sensor glucose and BST levels were simultaneously assessed for glycemic changes and when adequate hypoglycemia was reached during the combined pituitary function test. The CGM sensor glucose, BST, and serum glucose levels showed similar glucose trends in all three patients. A Bland–Altman analysis revealed that the CGM underestimated the BST values by approximately 9.68 mg/dL, and a Wilcoxon signed-rank test showed that the CGM and BST measurements significantly differed during the stimulation test (*p* = 0.003). Nevertheless, in all three cases, the CGM sensor mimicked the glycemic variability changes in the BST reading and assisted in monitoring appropriate hypoglycemia nadir. Thus, CGM can be used as a safe aid for clinicians to use during insulin tolerance tests where critical hypoglycemia is induced.

## 1. Introduction

Hypopituitarism is a chronic endocrine illness caused by the partial or total loss of hormone production in the anterior and posterior pituitary glands. Various diseases, including brain neoplasms, pituitary ischemic necrosis, and other systemic disorders; traumatic brain injury; and radiation to the brain can cause hypopituitarism. An impaired pituitary gland function may result in deficiencies in the levels of adrenocorticotropic thyrotropin, gonadotropin, prolactin, antidiuretics, growth hormone (GH), or a combination of some of these deficiencies. Patients with multiple pituitary hormone deficiencies require lifelong hormone replacement therapy; therefore, an accurate diagnosis is crucial. A diagnosis can be made by measuring the basal hormone levels and performing stimulation tests to determine the deficient hormones. A GH deficiency during childhood often leads to a deficiency in adulthood, thus requiring a re-evaluation and GH replacement after childhood [1]. Furthermore, an adult GH deficiency is a heterogeneous disorder involving dyslipidemia, low bone mass, and increased resistance and fat mass; GH replacement in adulthood has been associated with an improved lipid profile [2,3,4]. Although various stimulation tests are available, the insulin tolerance test remains the gold-standard method for diagnosing GH deficiencies [1,5].

The combined pituitary function test, often called the cocktail test, is a dynamic test that is used to evaluate the anterior pituitary gland. It involves the simultaneous injection of insulin, gonadotropin-releasing hormone (GnRH), and thyrotropin-releasing hormone (TRH) to assess the levels of cortisol, GH, prolactin, thyroid-stimulating hormone (TSH), luteinizing hormone (LH), and follicle-stimulating hormone (FSH). In insulin tolerance tests, insulin is injected to induce adequate symptomatic hypoglycemia (a serum blood glucose level <40 mg/dL or <45 mg/dL with a 50% reduction from baseline). The induced stress, which, in this case, is hypoglycemia, leads to an increase in the GH and adrenocorticotropic hormone (ACTH) levels, resulting in the elevation of cortisol levels [6]. An insufficient increase in the GH and cortisol levels after stimulation confirms the diagnosis of a GH deficiency and/or adrenal function insufficiency. However, this test is often very tedious, requiring close medical supervision, and the possibility of life-threatening hypoglycemic events is unpredictable.

A continuous glucose-monitoring (CGM) system includes a medical device that measures the interstitial fluid glucose levels using a subcutaneous glucose sensor probe. Glucose readings are wirelessly transmitted at set intervals (1–5 min, depending on the device) to a reader or a smartphone application, enabling the near real-time glucose monitoring of patients. CGM can not only read on-demand glucose levels, but also monitor glycemic variability and detect hypoglycemic events in patients with diabetes. Studies have shown that CGM improves glycemic control in adolescents and adults with diabetes mellitus, and the devices are now widely used even in pediatric populations [7,8]. Furthermore, various efforts are being made to utilize this technology in non-traditional fields because of its ability to easily measure serum glucose levels and detect hyperglycemic and/or hypoglycemic events [9]. The application of CGM in healthy individuals has suggested that the device is effective as a screening tool for glucose regulation as well as lifestyle and athletic performance optimization [10]. Recently, CGM demonstrated usefulness in identifying asymptomatic hypoglycemia events following gastric bypass surgery in non-diabetic populations [11]. However, to the best of our knowledge, CGM was not previously used in stimulation tests where hypoglycemia is induced.

Herein, we describe three cases in which CGM was successfully used simultaneously with blood sugar tests (BSTs) during the cocktail test for the better detection and induction of hypoglycemia.

## 2. Materials and Methods

**Figure 1:**
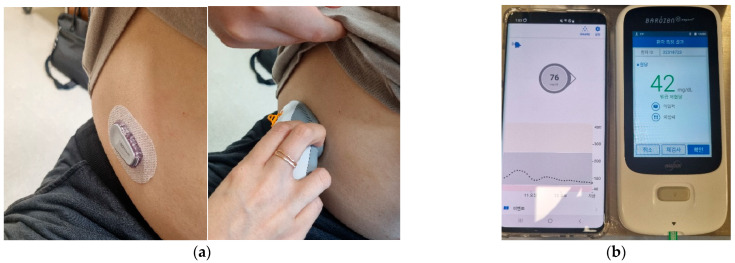
(**a**) A Dexcom G6 sensor and transmitter were applied onto the patients’ abdomen or upper arm; (**b**) an image of the CGM mobile application screen and BST sample measurement during the combined pituitary function test.

This study was supported by Dexcom, which provided the team with glucose sensors and readers. The factory-calibrated Dexcom G6 CGM (Dexcom Inc., San Diego, CA, USA) was used as the glucose-monitoring device. Dexcom G6 is a small, wearable sensor that has a 10-day lifetime and can track real-time glucose readings automatically every 5 min. The device did not require calibration, and previous studies have reported an overall good accuracy with a mean absolute relative difference (MARD) of 10% [12]. All three participants were diagnosed with multiple pituitary hormone deficiencies during childhood and required a re-evaluation before transitioning into treatment in adulthood. Each patient received a CGM device 5–7 days before admission and was provided with device-related training by the nursing staff (Figure 1a).

**Figure 2:**
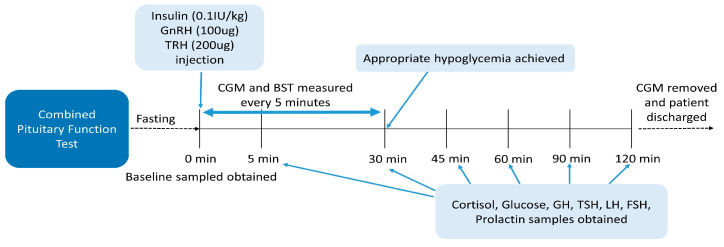
Scheme of the combined pituitary function test protocol. Patients fasted overnight. A combination of insulin, gonadotropin-releasing hormone (GnRH), and thyrotropin-releasing hormone (TRH) was injected, and baseline samples were obtained at 0 min.

On admission, the patients fasted overnight. A combination of insulin (0.1 IU/kg Novorapid; Novo Nordisk Pharma, Seoul, Korea), GnRH (100 ug Decapeptyl; Ferring Pharmaceuticals, Seoul, Korea), and TRH (200 ug Preline; Korea United Pharm, Seoul, Korea) was administered on the day of the test. Serum samples of cortisol, ACTH, GH, TSH, sex hormone (estrogen/testosterone), LH, and FSH were obtained at baseline. The levels of cortisol, glucose, GH, TSH, LH, FSH, and prolactin were measured at 30, 45, 60, 90, and 120 min after appropriate hypoglycemia was achieved (Figure 2). Appropriate hypoglycemia was defined as the achievement of symptomatic hypoglycemia and a BST level below 45 mg/dL. Additional insulin (0.05 IU/kg) was administered if the patient did not reach appropriate hypoglycemia. The CGM sensor glucose levels and BST changes were simultaneously recorded during the test (Figure 1b). The hypoglycemia alarm for the CGM sensor was set to 45 mg/dL. Medical staff were on site throughout the test to monitor the vital signs and record the hypoglycemia symptoms. Following the hypoglycemia nadir, the patient was given a cup of orange juice or received on-demand glucose rescue with an IV dextrose bolus administration when he/she showed significant symptoms of lethargy or drowsiness or when the glucose level failed to rise. A GH deficiency was diagnosed when the GH response was <5 ng/mL. Hypogonadism was diagnosed when an inadequate LH (<2–3 times higher than the baseline or peak LH level <5 IU/L) and FSH (<1.5–2 times higher than the baseline or peak FSH level <5 IU/L) response was evident. A peak cortisol level <18 ug/dL was defined as an ACTH deficiency. Finally, thyroid dysfunction was diagnosed when the peak TSH level failed to rise by >5–10 mIU/L or 2.5 times the baseline level and the prolactin increments were <2.5 times the baseline level [1,13,14].

None of the participants showed device-related adverse events, and the CGM device was removed prior to discharge.

## 3. Case Presentation

### 3.1. Case 1

**Figure 3:**
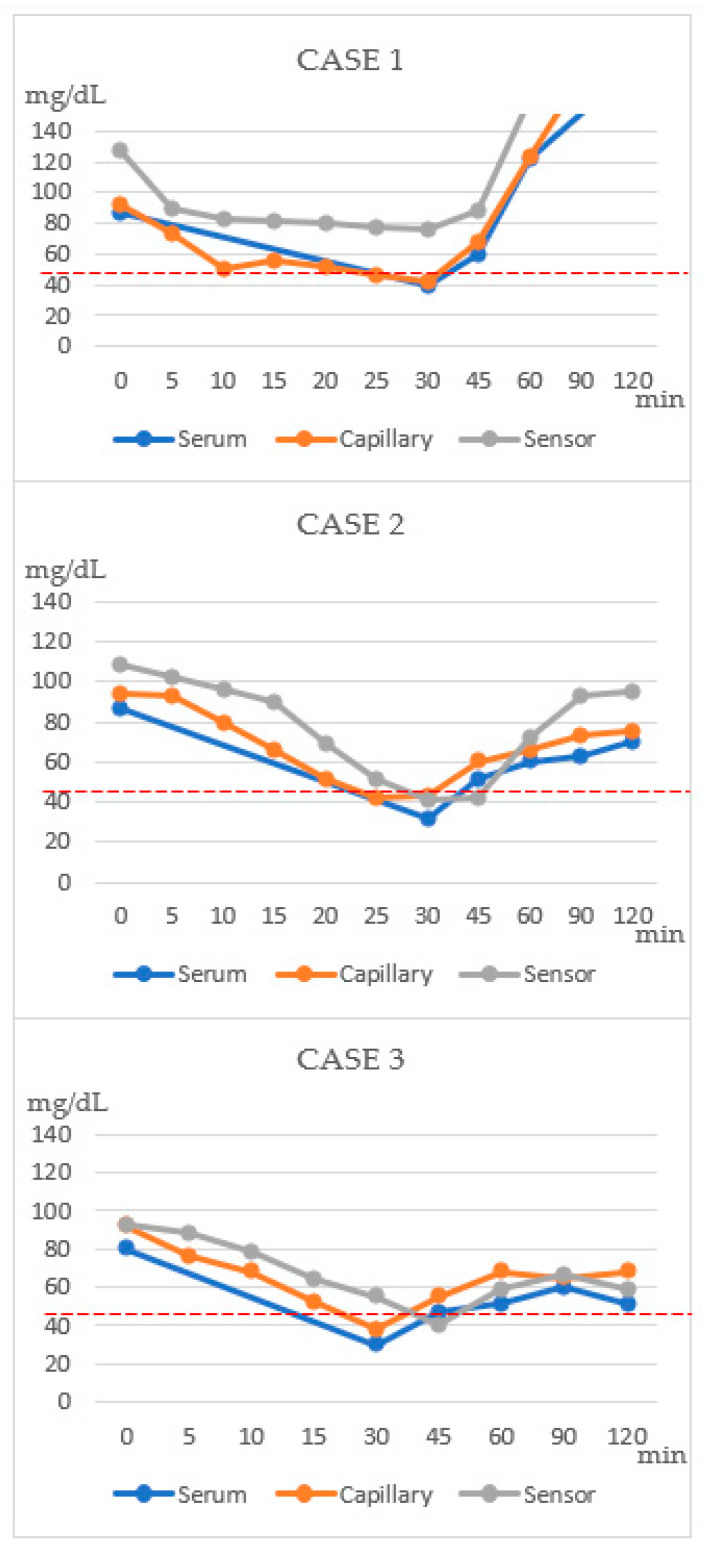
Glucose trends during the combined pituitary function test. Serum: serum glucose level; capillary: capillary blood sugar test (BST) level; and sensor: CGM sensor-measured glucose level. BST and CGM measurements were taken every 5 min until hypoglycemia was reached. Serum glucose, BST, and CGM measurements were taken at 0, 30, 45, 60, 90, and 120 min after stimulation.

A 24-year-old male was diagnosed with pilocytic astrocytoma of the brain at the age of 12 years and underwent a craniotomy and tumor removal. Following surgery, the patient received chemotherapy and was referred to our pediatric endocrinology clinic for evaluation. Since then, he has been diagnosed with multiple pituitary hormone deficiencies, including a GH deficiency, secondary hypogonadism, secondary hypothyroidism, and diabetes insipidus. The patient has received the corresponding treatments, including GH replacement therapy. At the age of 24 years, the patient was admitted for a re-evaluation of multiple hormone deficiencies. His height, weight, and body mass index (BMI) were 183 cm, 94.7 kg, and 28.3 kg/m^2^, respectively. During the cocktail test, the patient showed symptoms of hypoglycemia—sweating, lightheadedness, and mild tremors—20 min after insulin administration. Appropriate hypoglycemia was achieved 30 min after the insulin administration, and the remaining tests were performed. The patient’s vital signs were stable, he remained symptom-free throughout the study, and then he was discharged from the hospital. The glucose trend of the patient is shown in Figure 3 and the results of the combined pituitary function test are presented in Table 1.

### 3.2. Case 2

A 23-year-old female was diagnosed at the age of 11 years with an infundibular mass, which was possibly a germinoma of the brain. As the mass showed no progression, her neurosurgeon decided not to proceed with a surgical intervention, and the mass remained stable throughout the following years. At the age of 14 years, the patient showed a decreased rate of growth and low levels of insulin-like growth factor I, and she was diagnosed with a GH deficiency through a stimulation test. However, the patient and her family decided not to receive GH replacement therapy through a shared decision-making process because the mass had not been removed. Over the years, the patient was diagnosed with diabetes insipidus and primary hypogonadism; she had been receiving subsequent treatments. At the age of 23 years, the patient was admitted to our hospital for a re-evaluation for multiple hormone deficiencies. Her height, weight, and BMI were 160 cm, 72 kg, and 28 kg/m^2^, respectively. The patient experienced lightheadedness and dizziness 30 min after insulin administration when the glucose levels, according to the CGM device and the BST, were <45 mg/dL (41 and 43 mg/dL, respectively; Figure 3). After completing the stimulation test, the patient recovered without further complications.

### 3.3. Case 3

In 2010, after being diagnosed with craniopharyngioma at the age of 10 years, the patient underwent an endoscopic transsphenoidal-approach tumor removal and received radiation therapy following the surgery. The patient was then diagnosed with a GH deficiency and received GH treatment, as well as treatments for adrenal insufficiency, diabetes insipidus, hypothyroidism, and hypogonadism. At the age of 22 years, the patient was admitted to our hospital for a multiple hormone deficiency re-evaluation. Upon admission, the patient’s height, weight, and BMI were 163.6 cm, 58.5 kg, and 21.8 kg/m^2^, respectively. Twenty minutes after injection with the stimulating hormones, the patient experienced dizziness, sweating, and appropriate hypoglycemia (CGM: 55 mg/dL; BST: 38 mg/dL; Figure 3).

## 4. Results

The combined pituitary function test confirmed the diagnosis of multiple pituitary hormone deficiencies (MPHDs) in all three patients (Table 1). The test results of patient 1 showed an adult GH deficiency with hypogonadotropic hypogonadism and secondary hypothyroidism. Upon the diagnosis, the patient was recommenced on GH therapy and resumed the administration of the previous medications for multiple pituitary hormone deficiencies. Patient 2 showed no GH response (pGH: 1.08 ug/L; normal range: >10 ug/L), and she was diagnosed with an adult GH deficiency (Table 1). As the patient had been receiving estrogen replacement, her androgen levels were within the normal range, and the thyroid hormone levels showed a normal response. The patient did not undergo surgery to remove the brain mass. Hence, she decided not to receive adult GH replacement therapy and to continue the previous treatments for diabetes insipidus and hypogonadism. The results of patient 3 showed multiple inadequate hormone responses, and the patient was diagnosed with a GH deficiency, hypogonadotropic hypogonadism, hypothyroidism, and adrenal insufficiency (Table 1). The patient was recommenced on GH replacement therapy upon discharge and administered hydrocortisone, testosterone, and a thyroid hormone treatment.

**Figure 4:**
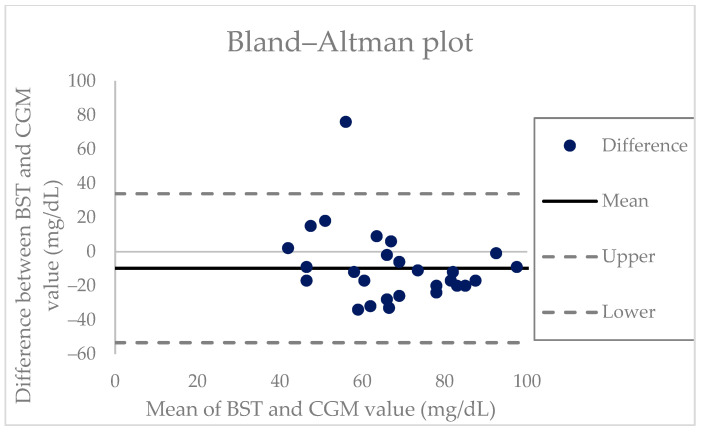
The Bland–Altman plot comparing CGM and BST measurements. The mean difference in all the readings is noted by a bold, black, and solid line with an additional reference line noted at zero. The dashed lines represent the standard deviation and provide an estimate of 95% differences. BST, blood sugar test; CGM: continuous glucose monitoring.

To examine the agreement between the CGM and BST values, a Bland–Altman plot was constructed (Figure 4) [15]. The average difference between the CGM and BST measurements was −9.68 and the limits of agreement were between −53.3 and 33.9. The Wilcoxon signed-rank test showed a significant difference between the two measurements (Z = −2.93, *p* = 0.003).

## 5. Discussion

Careful glucose monitoring is crucial during combined pituitary function tests where insulin is administered to induce a possibly dangerous hypoglycemia in order to evaluate the pituitary gland. For its ability to capture near real-time glucose variability, CGM systems are being increasingly experimented with in various clinical settings other than those involving diabetes. We report a novel attempt to utilize CGM as a safe glucose-monitoring aid in stimulation tests where critical hypoglycemia is induced.

The combined pituitary function test is an old-fashioned examination that is often tedious to perform. The likelihood of the occurrence of life-threatening hypoglycemia requires close medical supervision by a physician during testing. There are no established guidelines for combined pituitary function testing and the frequency of changes in glucose levels [16]. Although the insulin tolerance test is often difficult to replicate, and the insulin response may be unpredictable, it is still the standard test for GH deficiencies [1]. Hypoglycemia triggers symptoms such as anxiety, sweating, tachycardia, and neurological responses such as tingling and faintness [17]. In some studies, the degree of hypoglycemia was much lower than the targeted blood glucose level in many patients [18]. Thus, although rare, hypoglycemia may result in neuroglycopenia and cause seizures and altered consciousness in some susceptible subpopulations. In addition, obese patients with insulin resistance may fail to achieve adequate serum hypoglycemia with a standard insulin dose. They may require the administration of additional higher insulin doses, which, in turn, increases the risk of delayed hypoglycemia. Moreover, nadir timing is unpredictable. Although the capillary glucose level is widely used as a quick and easy parameter to assess the glucose levels, some studies have reported a worrisome discrepancy between capillary glucose and serum glucose levels [19]. Relying solely on the capillary BST may hinder the accuracy of the test. Importantly, frequent skin-prick testing is invasive. Nevertheless, as serum glucose testing results take a longer time than BST results, physicians must rely on frequent capillary BSTs during testing to monitor hypoglycemia and safely complete the test.

In 1979, Karam et al. first utilized a CGM linked to a continuous insulin infusion system during the surgical management of a patient with insulinoma [20]. Remarkable advancements have been made over the years, where modern CGM devices consist of wearable sensors as small as two stacked U.S. pennies, and information can be wirelessly transmitted to a cell phone application. The accuracy of CGM differs according to the report, but it has been well validated in many studies [12,21]. The MARD value, which is the average of the absolute errors between all CGM values, is often used. The overall MARD has been reported to be reliable in most commercially available CGM systems at 8–10%, and 10% for Dexcom G6 [12,22]. Nowadays, the International Society for Pediatric and Adolescent Diabetes, the American Diabetes Association, and the National Institute for Health and Care Excellence recommend CGM as the preferred option for glucose monitoring in their consensus guidelines for children and adults with diabetes mellitus [23,24,25].

Recently, efforts have been made to utilize this device for glucose monitoring in conditions other than diabetes. In one study, CGM was used during acute lymphoblastic leukemia maintenance therapy to characterize the pattern of hypoglycemia and validate the benefit of cornstarch for hypoglycemia associated with 6-MP treatment [26]. In addition, a recent study revealed that CGM technology is highly convenient for monitoring glucose levels, even in inpatient hospital settings [27]. Several studies have also used CGM in neonatal intensive care units to reduce the frequency of the painful procedures of skin-prick tests; CGM has been shown to reduce the incidence of hypoglycemic events and detect hidden hypoglycemic events [28,29]. Others have reported CGM to be a feasible tool for intraoperative glucose monitoring, providing better glycemic trends than those obtained from individual blood glucose readings [30]. CGM has proven beneficial in settings with frequent hypoglycemia by detecting a decreasing trend earlier than serum glucose checks. Another study found that CGM during an oral glucose tolerance test in healthy young adults was a possible alternative to sampling serum glucose concentrations [31]. Although many studies have applied CGM as a glucose-monitoring device outside the setting of diabetes, none have utilized the technology in stimulation tests where severe hypoglycemia is induced.

Taking advantage of the ability of CGM to capture the changes in glucose levels in near real-time, the present study applied CGM to three patients undergoing multiple pituitary hormone tests, including the insulin tolerance test. The goal of the test is to achieve appropriate hypoglycemia with a serum blood glucose level <40 mg/dL; hence, monitoring the glucose level is essential. In our study, the CGM sensor detected glucose trends that correlated well with the decline in the actual serum glucose levels. In all three patients, the CGM level showed a decline with a decrease in the capillary glucose level. All the patients safely completed the test. The Bland–Altman plot shows the difference between the CGM and BST glucose measurements, plotted against their means [15]. Overall, the CGM underestimated the BST values by approximately 9.68 mg/dL, and wide limits of agreement were observed. This finding is consistent with those of previous studies, which reported that CGM displayed lower readings than those observed for the reference during hypoglycemic events [32]. The high random errors may be due to both the CGM device and patient-specific factors; however, only 30 matched points were included in the plot, and a further analysis with larger data points is needed. A statistical analysis using the Wilcoxon signed-rank test revealed that the CGM and BST measurements showed significantly different values during the stimulation test (*p* = 0.003). However, again, the data were limited to less than 30 set points, and the statistical difference did not seem to be of high significance in this study. The study protocol only required BST sampling until appropriate hypoglycemia was achieved (which was usually within 30 min into the test). Thus, we were unable to collect CGM and BST value data beyond the test protocol in the studied patients, as it was unnecessary. Nevertheless, the aim of this case series was not to show whether the CGM value can accurately match the BST measurement in a stimulation test. Rather, we attempted to show the usefulness of the CGM device in monitoring the glucose trend in the stimulation test where hypoglycemia induction is required so that the frequency of skin-prick tests can be minimized. Although the CGM sensor may not fully reflect the serum glucose level, it does show the glucose trend in real time. In addition, CGM has the advantage of sparing the patients from the pain associated with frequent BST checks. With the use of the device, physicians can monitor the decreases in glucose levels experienced by the patients and detect hypoglycemia with minimal BST checks. A sharp decline in the CGM levels may indicate the need for a skin-prick test to confirm adequate hypoglycemia. Despite all three patients being adults, the device should hold more value in pediatric patients for whom less invasive monitoring is often favored. By providing a continuous trend of glucose variability in real time, CGM serves as a highly attractive alternative to frequent skin-prick tests for monitoring glucose levels in stimulation tests and offers an improved quality of life.

This study has some limitations. First, the CGM did not show a 100% correlation with the serum glucose levels, which can be misleading. Hence, the accuracy of CGM in detecting hypoglycemia remains controversial. Previous studies have reported that CGM is less accurate in hypoglycemic ranges [12,33]. The MARD score is higher in hypoglycemic values (50–70 mg/dL) than in the euglycemic state of patients with diabetes, and its accuracy is lower in patients without diabetes [34]. As this study aimed to detect hypoglycemic events in patients without diabetes, the CGM values may have been less accurate than the actual serum glucose levels. Nevertheless, CGM was not used in this study for diagnosing diabetes mellitus, but rather for monitoring the hypoglycemic trend in patients in whom a critically low glucose level was induced. Second, a time lag between the change in plasma glucose levels and the interstitial measurement of glucose by CGM may have existed, and a previous study has indicated that CGM is less reliable when rapid changes in glucose levels are induced [35]. Others have shown that CGM may overestimate glucose levels up to 40 mg/dL during periods of a rapid decline in glucose levels [28]. Although the G6 CGM system does not require a calibration period, studies have shown that calibration may affect the accuracy [12]. Third, the access to CGM is limited. The device can be costly, and in most countries, CGM has not yet been licensed for uses other than glucose monitoring in patients with type 1 diabetes. In addition, owing to its designed use for several days to weeks, the per-unit costs of CGM sensors are much higher than those of BST strips. Therefore, implementing the device as an assistant tool in stimulation tests may not be cost-effective. Fourth, this case report is only limited to our experience with three cases of patients; thus, larger-scale studies are needed to validate CGM use in stimulation tests where hypoglycemia induction is required. Nevertheless, the CGM and BST measurements showed adequate hypoglycemia values in all three patients in our study and successful stimulation tests were performed. Although the devices may be costly, our case report showed that a CGM application may protect patients from painful skin-prick BST tests and provide physicians with a visible glucose-level trend. Nevertheless, further investigations with more participants and pairwise analyses are required.

In conclusion, CGM technology has shown significant improvements and holds value in applications beyond diabetes care. Although many attempts have been made to utilize CGM in various fields, its use in the combined pituitary hormone stimulation test has not yet been documented. We attempted to demonstrate the use of the CGM technology beyond the field of diabetes by reporting its use in the combined pituitary hormone stimulation test where hypoglycemia is intentionally induced. Further studies are warranted for the future application of the CGM technology to provide patients and physicians with a pain-free and safe option by implementing an alternative for monitoring the glucose decline during hormone stimulation tests.

## Figures and Tables

**Table 1:** Results of the combined pituitary function tests.

	Case 1		Case 2		Case 3	
	Baseline	Peak	Baseline	Peak	Baseline	Peak
Insulin tolerance test						
Glucose (mg/dL)	48		87		80	
GH (ng/mL)	0.08	<0.04	0.06	1.08	<0.04	<0.04
Cortisol (ug/dL)	20.05	35.25	10.42	22.57	0.22	0.38
IGF-I (ng/mL)	121		61.12		133	
TRH stimulation test						
TSH (mIU/L)	1	3.8	2.56	13.92	<0.06	<0.06
fT4 (ng/dL)	1.19		0.99		1.24	
Prolactin (ng/mL)	11.58	22.05	4.41	31.25	1.04	2.82
GnRH stimulation test						
LH (mIU/mL)	0.98	4.06	2.07	11.01	1.08	1.79
FSH (mIU/mL)	1.43	4.92	1.98	6.92	0.13	0.34
Testosterone (ng/mL)	0.47	-	-	-	2.44	-
E2 (pg/mL)	-	-	245.7	-	-	-

E2, estradiol; FSH, follicle-stimulating hormone; fT4, free thyroxine; GH, growth hormone; GnRH, gonadotropin-releasing hormone; IGF-I, insulin growth factor-I; IGFBP-3, insulin-like growth factor-binding protein-3; LH, luteinizing hormone; TRH, thyrotropin-releasing hormone; TSH, thyroid-stimulating hormone.

## Data Availability

The data that support the findings of this study are available from the corresponding author upon reasonable request.
